# Characterization of Two *AGAMOUS*-like Genes and Their Promoters from the *Cymbidium faberi* (Orchidaceae)

**DOI:** 10.3390/plants12142740

**Published:** 2023-07-24

**Authors:** Jiayi Li, Ling Wang, Xiangjian Chen, Lingtian Zeng, Yalan Su, Zhixiong Liu

**Affiliations:** College of Horticulture and Gardening, Yangtze University, Jingzhou 434025, China; 2021710820@yangtzeu.edu.cn (J.L.); 201971415@yangtzeu.edu.cn (L.W.); 202071696@yangtzeu.edu.cn (X.C.); 202072800@yangtzeu.edu.cn (L.Z.); 2022710854@yangtzeu.edu.cn (Y.S.)

**Keywords:** *AGAMOUS*, *Cymbidium faberi*, flower development, gynostemium, orchid

## Abstract

*Arabidopsis AGAMOUS* (*AG*) play roles in determining stamens’ and carpels’ identities, floral meristem determinacy, and repression of the A-function. Gynostemium fused by stamens and carpels is a characteristic reproductive structure in orchid flowers, which shows a considerable difference from the reproductive organs of eudicots and other monocot species. The molecular basis of orchid gynostemium development remains largely unknown. Here, we report the identification and functional characterization of two *AG*-like genes, *CyfaAG1* and *CyfaAG2*, and their promoters from *C. faberi*. Both *CyfaAG1 and CyfaAG2* are highly expressed in the anther cap, gynostemium, and ovary. Ectopic expression of *CyfaAG1 and CyfaAG2* promotes early flowering of wild-type *Arabidopsis.* Moreover, ectopic expression of *CyfaAG1* completely rescues floral defects in the *Arabidopsis ag-1* mutant, while ectopic expression of *CyfaAG2* only completes filament and carpel development. Our findings suggest that *CyfaAG1* acts as an evolutionarily conserved C-function gene in determining reproductive organ identity and mediating floral meristem determinacy. *CyfaAG2* redundantly mediates the C-function in floral meristem determinacy and gynostemium development. Our results provided more details to understand how the C-class function has been partitioned in orchids, and the roles of two *AG* orthologs in regulating gynostemium development in *C. faberi*.

## 1. Introduction

*Cymbidium faberi* Rolfe is a very popular potted orchid and has been cultivated for centuries in China, Japan, and Korea [1]. As a Chinese traditional famous flower, numerous varieties with diverse flower phenotypes have been developed during several thousand years of domestication. However, the mechanism for regulating the floral organ morphogenesis of *C. faberi* remains unclear. Previous studies suggested that different homeotic MADS transcription factors work together to specify the identities of different types of floral organs during flower development [2,3]. In *Arabidopsis*, the C-class MADS-box transcription factor *AGAMOUS* (*AG*) plays a key role in regulating stamen and carpel identities as well as meristem determinacy [4]. Moreover, most *AG* orthologs from other eudicots retain functional conservation in determining reproductive organ identity and meristem determinacy during flower development [5]. However, the C-class function has been partitioned among monocots, since the *AG* lineage is further duplicated within this taxa [6,7,8,9]. In rice, two C-class genes, *OsMADS3* and *OsMADS58*, have diversified with separate functions: *OsMADS3* is crucial for stamen identity and *OSMADS58* is crucial for floral meristem determinacy and carpel morphogenesis [6,10]. Moreover, both genes share redundancy in the regulation of stamen and carpel identities [6,11,12]. The C-class function has also been partitioned in maize, *ZMM2* and *ZMM23* are orthologs of *OsMADS3* and are crucial for organ (stamen and carpel) identity, while *ZAG1* is an ortholog of *OsMADS58* and crucial for floral meristem determinacy [13,14]. In orchids, the C-class gene *DOAG1* from *Dendrobium* was proved to specify reproductive organs, mediate perianth development, and regulate floral meristem determinacy [7]. In *C. sinense* (Orchidaceae), two *AG*-like genes *CsAG1* and *CsAG2* also showed functional redundancy and divergence. *CsAG1* expressed in the tepals, lips, and gynostemium, and was involved in reproductive organ development, while *CsAG2* was highly expressed in the gynostemium but its function remained unclear [9]. In addition, an *AG*-like gene, *CeMADS1*, may have a pivotal C function in reproductive organ development in *C. ensifolium*, while the function of another C-class *AG*-like gene, *CeMADS2,* remains unclear [15].

**Figure 1:**
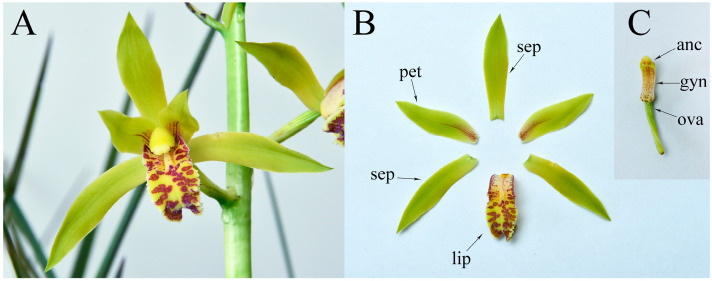
Flower of *Cymbidium faberi*. (**A**) Flower of *C. faberi*; (**B**) perianth (sepal, petal and lips) of *C. faberi*; (**C**) anther cap, gynostemium, and ovary of *C. faberi*. Sepals (sep), petals (pet), lips (lip), anther cap (anc), gynostemium (gyn), ovary (ova).

The gynostemium, a column of fused stamens and carpels, is a characteristic reproductive structure in the orchid flower, which shows a considerable difference from the reproductive organ of eudicots and other monocot species. Moreover, two *AG*-like genes resulted from a duplication event predating the divergence of Orchidaceae species [9]. Although one *AG* lineage gene harboring C function was identified in *Dendrobium*, *C. sinense*, and *C. ensifolium* [7,9,15], the function of another AG lineage gene should be further extensively explored. In this study, we isolated two C-class genes, *CyfaAG1* and *CyfaAG2*, and their promoters from *C. faberi*. The flower of *C. faberi* consists of three whorls of floral organs, with three sepals in whorl 1, two petals and a lip in whorl 2, and gynostemium-fused stamens and carpels in whorl 3 (Figure 1). In addition, we also characterized the functions of both genes and promoters. Our results provided more details to understand how C-class function has been partitioned in orchid, and the functions of two *AG*-like genes involved in the gynostemium development of *C. faberi*.

## 2. Results

### 2.1. Isolation and Characterization of CyfaAG1 and CyfaAG2 from Cymbidium faberi

**Figure 2:**
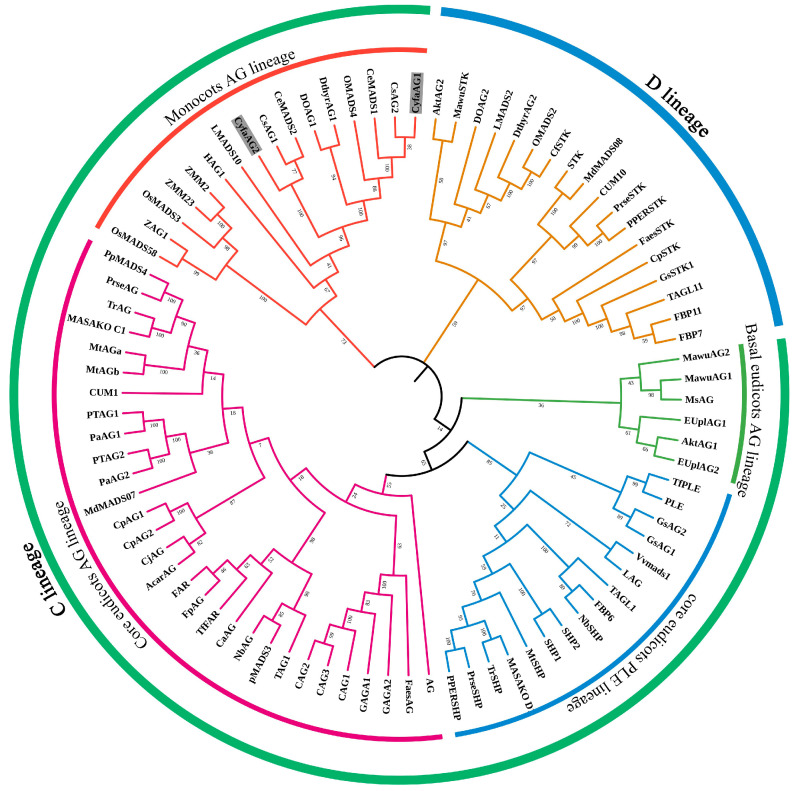
Phylogenetic tree of CyfaAG1, CyfaAG2, and other AG subfamily transcription factors from different clades of angiosperms.

The 987 bp *CyfaAG1* cDNA contains a 702 bp ORF (open reading frame, ORF) encoding 233 amino acids (aa) (Genbank accession number: MH917913.1), while the 997 bp *CyfaAG2* cDNA contains a 705 bp ORF (open reading frame, ORF) encoding 234 aa (Genbank accession number: MH917914.1). However, the transcription factors encoded by both genes showed only 85.90% identity. Phylogenetic tree analysis grouped CyfaAG1 and CyfaAG2 into monocots’ AG lineage (Figure 2), and the genes were designated as *CyfaAG1* (*Cymbidium faberi AGAMOUS*) and *CyfaAG2*, respectively.

### 2.2. Isolation and Identification of CyfaAG1 and CyfaAG2 Promoters from Cymbidium faberi

A 1.4 kb *CyfaAG1 promoter* (*pCyfaAG1*) (−1207/+174) (Genbank accession number: OM032618.1) and a 1.6 kb *CyfaAG2 promoter* (*pCyfaAG2*) (−1473/+148) (Genbank accession number: OM032619.1) were separately isolated from *C. faberi*. The putative transcription start site and regulatory cis-elements of *pCyfaAG1* and *pCyfaAG2* are separately displayed in Figures S1 and S2. The *pCyfaAG1* has a critical element (CArG-box) (−1203/−1194) for MADS-box transcription factor binding to regulate flower development, while *pCyfaAG2* has two CArG-boxes (−1058/−1049 and +141/+150) [16]. In addition, *pCyfaAG1* has five anther-specific elements (POLLEN1LELAT52-box) and five pollen-specific elements (GTGANTG10-box) [17,18], and *pCyfaAG2* contains six POLLEN1LELAT52-boxes and a GTGANTG10-box, which suggests that *CyfaAG1* and *CyfaAG2* may work together to regulate gynostemium development in *C. faberi*. Moreover, three conserved binding sites (AACAAA-/TTTGTT- motif) for floral homeotic APETALA2 transcription factor are separately found in *pCyfaAG1* and *pCyfaAG2* [19]. In addition, some gibberellin-responsive elements (WRKY71OS-box and CAREOSREP1-box) [20,21], and abscisic acid-responsive elements (MYB1AT-box) [22] are separately found in *pCyfaAG1* and *pCyfaAG2*. Moreover, auxin-responsive elements (D2GMAUX28-box) and gibberellin-responsive elements (PYRIMIDINEBOXOSRAMY1A-box) are found in *pCyfaAG1* and *pCyfaAG2*, respectively [23,24]. In addition, cytokinin-responsive element ARR1AT motif is also found in *pCyfaAG2* [25]. These data suggest that gibberellin (GA) and abscisic acid (ABA) signaling pathways may also directly regulate the floral homeotic genes *CyfaAG1* and *CyfaAG2* expressions, and the auxin and cytokinin signaling pathways may regulate *CyfaAG1* and *CyfaAG2* expressions, respectively. Furthermore, several mesophyll-specific elements (CACTFTPPCA1-box) are lying at *pCyfaAG1* and *pCyfaAG2*, but two axillary bud-specific elements (SREATMSD-box) are found only in *pCyfaAG1*, which suggested that the corresponding gene expression may extend to bud, leaf, and rachis [26,27]. However, two recognizing and binding sites (CCAATBOX1) for the CONSTANS transcription factor to promote flowering are only found in *pCyfaAG1* [28]. These suggest that *pCyfaAG1* and *pCyfaAG2* may drive the corresponding gene to regulate flowering and floral organ development in different ways.

### 2.3. Deletion Analysis of pCyfaAG1 and pCyfaAG2 in Transgenic Arabidopsis

**Figure 3:**
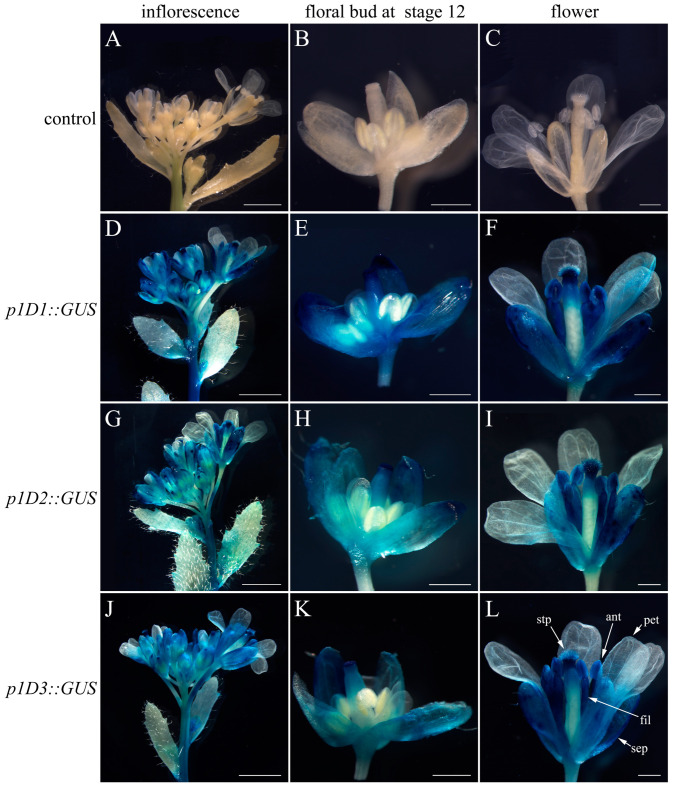
Deletion analysis of the *pCyfaAG1* promoter and histochemical GUS staining in the T1 generation transgenic *Arabidopsis*. (**A**) Wild-type *Arabidopsis* inflorescence; (**B**) stage 12 floral bud of wild-type *Arabidopsis* [29]; (**C**) wild-type *Arabidopsis* flower; (**D**) inflorescence of *p1D1*::*GUS* transgenic *Arabidopsis*; (**E**) stage 12 floral bud of *p1D1*::*GUS* transgenic *Arabidopsis*; (**F**) flower of *p1D1*::*GUS* transgenic *Arabidopsis*; (**G**) inflorescence of *p1D2*::*GUS* transgenic *Arabidopsis*; (**H**) stage 12 floral bud of *p1D2*::*GUS* transgenic *Arabidopsis*; (**I**) flower of *p1D2*::*GUS* transgenic *Arabidopsis*; (**J**) inflorescence of *p1D3*::*GUS* transgenic *Arabidopsis*; (**K**) stage 12 floral bud of *p1D3*::*GUS* transgenic *Arabidopsis*; (**L**) mature flower of *p1D3*::*GUS* transgenic *Arabidopsis*. Sepal (sep), petal (pet), anther (ant), filament (fil), stigmatic papillae (stp). Scale bars: (**A**,**D**,**G**,**J**) 2 mm; (**C**,**F**,**I**,**L**) 500 μm.

**Figure 4:**
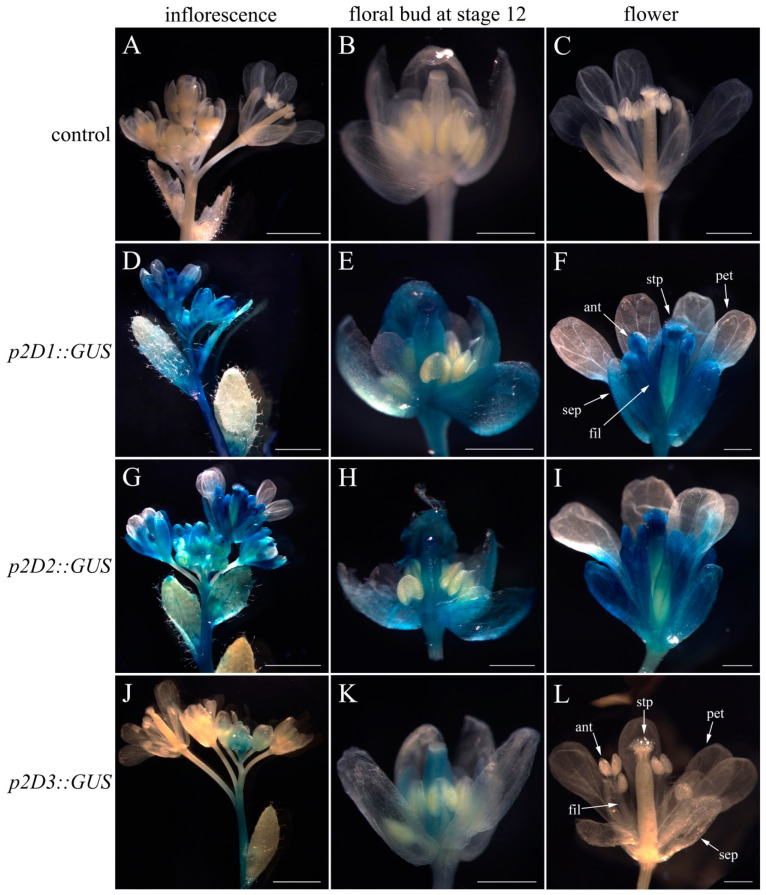
Deletion analysis of the *pCyfaAG2* promoter and histochemical GUS staining in the T1 generation transgenic *Arabidopsis*. (**A**) Wild-type *Arabidopsis* inflorescence; (**B**) stage 12 floral bud of wild-type *Arabidopsis* [29]; (**C**) wild-type *Arabidopsis* flower; (**D**) inflorescence of *p2D1*::*GUS* transgenic *Arabidopsis*; (**E**) stage 12 floral bud of *p2D1*::*GUS* transgenic *Arabidopsis*; (**F**) flower of *p2D1*::*GUS* transgenic *Arabidopsis*; (**G**) inflorescence of *p2D2*::*GUS* transgenic *Arabidopsis*; (**H**) stage 12 floral bud of *p2D2*::*GUS* transgenic *Arabidopsis*; (**I**) flower of *p2D2*::*GUS* transgenic *Arabidopsis*; (**J**) inflorescence of *p2D3*::*GUS* transgenic *Arabidopsis*; (**K**) stage 12 floral bud of *p2D3*::*GUS* transgenic *Arabidopsis*; (**L**) flower of *p2D3*::*GUS* transgenic *Arabidopsis*. Sepal (sep), petal (pet), anther (ant), filament (fil), stigmatic papillae (stp). Scale bars: (**A**,**D**,**G**,**J**) 2 mm; (**C**,**F**,**I**,**L**) 500 μm.

**Figure 5:**
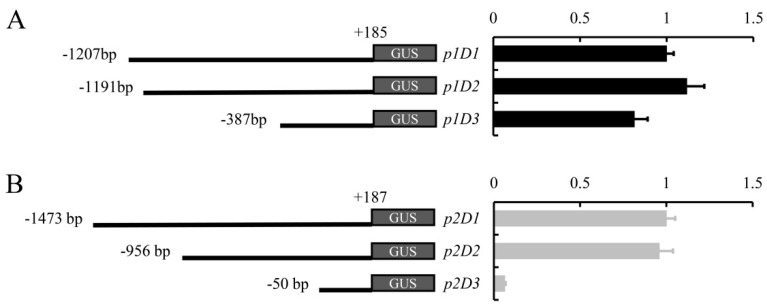
*GUS* expression in different transgenic *Arabidopsis* was separately confirmed by qRT−PCR with *actin* as the internal control. (**A**) *GUS* expression in *p1D1*::*GUS*, *p1D2*::*GUS,* and *p1D3*::*GUS* transgenic *Arabidopsis* was separately confirmed by qRT−PCR. (**B**) *GUS* expression in *p2D1*::*GUS*, *p2D2*::*GUS,* and *p2D3*::*GUS* transgenic *Arabidopsis* was separately confirmed by qRT−PCR.

A *GUS* reporter gene separately driven by a series of 5′ deletions fragments of *pCyfaAG1* and *pCyfaAG2* was assayed in transgenic *Arabidopsis* to analyze the regulatory effect of different regions of the corresponding promoter (Figure 3, Figure 4 and Figure 5). GUS staining was separately examined in the T1 generation of different deletion constructs. Moreover, GUS staining was obviously observed in the inflorescence and mature flower where sepal, stamen (filament and anther), stigma, and stigmatic papillae staining were intense, but was almost absent in petal of *p1D1*::*GUS*, *p1D2*::*GUS,* and *p1D3*::*GUS* transgenic *Arabidopsis* (Figure 3D,F,G,I,J,L). However, GUS staining suggested that *pCyfaAG1* drove *GUS* to extensively express in the sepal, filament, and gynoecium of stage 12 floral buds, but *GUS* expression was not observed in the anther and petal in *p1D1*::*GUS* transgenic *Arabidopsis* (Figure 3E) [29]. In addition, relatively weak GUS staining was separately observed in the sepal and gynoecium of stage 12 floral buds, but was almost absent in the petal and stamen of the stage 12 floral buds in *p1D2*::*GUS* and *p1D3*::*GUS* transgenic *Arabidopsis* (Figure 3H,K). In addition, GUS staining was obviously observed in the inflorescence and mature flower where sepal, stamen, and gynoecium were intense, but was absent in petal of *p2D1*::*GUS* and *p2D2*::*GUS* transgenic *Arabidopsis*, respectively (Figure 4D,F,G,I). Moreover, GUS staining was also observed in the sepal, filament, and gynoecia of stage 12 floral buds, but was absent in the anther and petal in *p2D1*::*GUS* and *p2D2*::*GUS* transgenic *Arabidopsis*, respectively (Figure 4E,H). However, GUS staining was only found in floral bud from appearance until stage 12 of *p2D3*::*GUS* transgenic *Arabidopsis* (Figure 4J). Furthermore, GUS staining was observed only in the gynoecium even in stage 12 of *p2D3*::*GUS* transgenic *Arabidopsis* (Figure 4K). A further deletion of the −956/−51 fragment from p2D2 (−956/+187) to produce p2D3 (−50/+187) caused obviously decreased GUS activity in transgenic *Arabidopsis* (Figure 4J,K and Figure 5B). These results suggested that the −956/−51 regions are capable of inducing *pCyfaAG2* promoter activity in the sepal, stamen, and gynoecia, and a 1143 bp region (−956/+187) of *pCyfaAG2* was sufficient for driving the *CyfaAG2* gene to regulate stamen and gynoecium development.

### 2.4. Expression Analysis of CyfaAG1 and CyfaAG2

**Figure 6:**
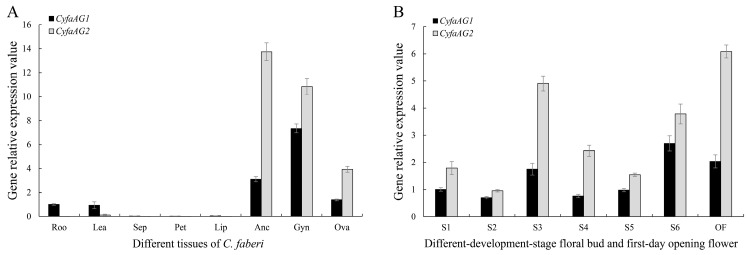
Expression patterns of *CyfaAG1* and *CyfaAG2*. (**A**) *CyfaAG1* and *CyfaAG2* expression in the root (Roo), juvenile leaf (Lea), sepal (Sep), petal (Pet), lip (Lip), anther cap (Anc), gynostemium (Gyn), and ovary (Ova) were detected by qRT−PCR with *Cyfaactin* as the internal control; (**B**) *CyfaAG1* and *CyfaAG2* expression in different development stages of the floral bud and at the first day of flower opening.

**Figure 7:**
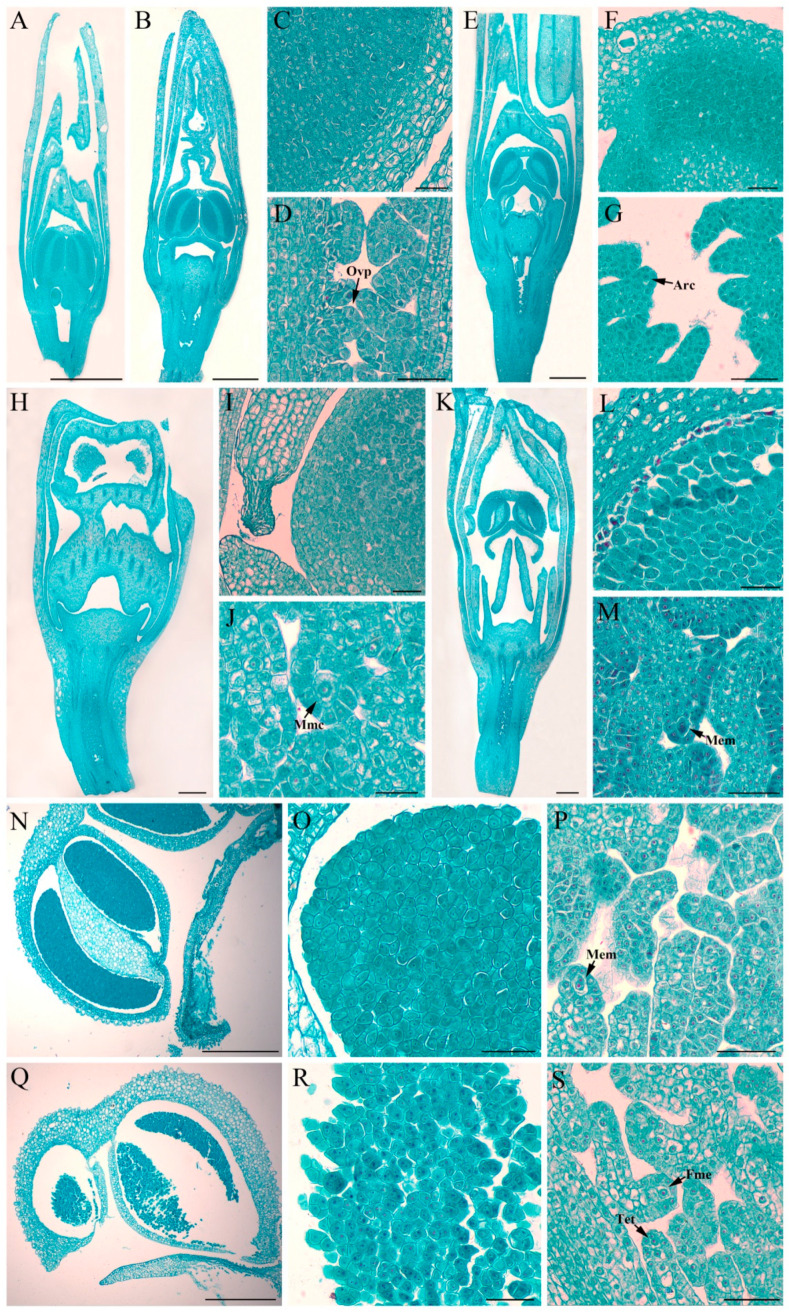
Morphology of different development stages of floral bud, anther, and ovary of *C. faberi*. (**A**) Stage 1 floral bud, about 5 mm in length; (**B**) stage 2 floral bud, about 8 mm in length (seasonal floral bud dormancy); (**C**) anther, enlargement of (**B**), microspore mother cell; (**D**) ovary, enlargement of (**B**), ovule primordia (Ovp) formation; (**E**) stage 3 floral bud, about 10 mm in length (breaking dormancy and further development); (**F**) anther, enlargement of (**E**), microspore mother cell beginning meiosis; (**G**) ovary, enlargement of (**E**), ovule primordia and archesporial cells (Arc); (**H**) stage 4 floral bud, about 15 mm in length; (**I**) anther, enlargement of (**H**), microspore mother cell beginning meiosis; (**J**) ovule, enlargement of (**H**), megaspore mother cell (Mmc); (**K**) stage 5 floral bud, about 20 mm in length; (**L**) anther, enlargement of (**K**), formation of microspore tetrads; (**M**) ovule, enlargement of (**K**), meiosis of megaspore mother cell (Mem); (**N**) anther, longitudinal section of stage 6 floral bud, about 25 mm in length; (**O**) anther, enlargement of (**N**), monokaryotic microspore within tetrads; (**P**) ovule, enlargement of (**N**), meiosis of megaspore mother cell; (**Q**) anther, longitudinal section of the first-day opening flower; (**R**) pollinium, enlargement of (**Q**), 2-cell pollen within mature tetrads of pollinium; (**S**) ovule, enlargement of (**Q**), linear tetrad (Tet) of megaspores, functional megaspore (Fme) formation. Scale bar: (**A**,**B**,**E**,**H**,**K**,**N**,**Q**) 1 mm; (**C**,**D**,**F**,**G**,**I**,**J**,**L**,**M**,**O**,**P**,**R**,**S**) 100 μm.

*CyfaAG1* was mainly expressed in root, leaf, anther cap, gynostemium, and ovary, while *CyfaAG2* showed relatively narrower expression zones and was expressed only in the anther cap, gynostemium, and ovary of *C. faberi* (Figure 6A). In addition, the expression level of *CyfaAG1* in the gynostemium was separately significantly higher than in the root, leaf, anther cap, and ovary (*p* < 0.05), while the expression level of *CyfaAG2* in the anther cap was significantly higher than in the gynostemium and ovary (*LSD*, *p* < 0.05) (Figure 4A). Moreover, the expression level of *CyfaAG2* in the anther cap or gynostemium was significantly higher than that of *CyfaAG1* in the anther cap or gynostemium, respectively (*p* < 0.05) (Figure 6A). *CyfaAG1* and *CyfaAG2* transcripts became detectable after primodium emergence of floral buds (Figure 6B and Figure 7A). During the seasonal floral bud dormancy by winter chilling (S2), both genes were expressed at low levels (Figure 4B). Moreover, with the dormancy broken and microspore mother cell beginning meiosis, the expressions of *CyfaAG1* and *CyfaAG2* increased obviously (Figure 6B and Figure 7E,F). Furthermore, *CyfaAG1* expression reached a peak at monokaryotic microspore within tetrads (S6), while *CyfaAG2* expression reached a peak at 2-cell pollen within mature tetrads of pollinium at the first day of flower opening (Figure 6B and Figure 7N,O,Q,R). However, *CyfaAG2* expression was separately significantly higher than that of *CyfaAG1* in S3 and of the first-day opening flower (*LSD*, *p* < 0.05) (Figure 6B).

### 2.5. Ectopic Expression of CyfaAG1 and CyfaAG2 in Arabidopsis ag-1 Mutant

**Figure 8:**
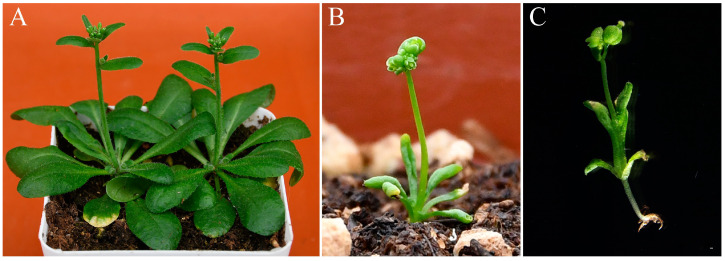
Early flowering phenotypes of *35S::CyfaAG1* and *35S::CyfaAG2* under wild-type background. (**A**) mature wild-type *A. thaliana* with 10–12 rosette leaves stage and normal raceme. (**B**) early flowering *35S::CyfaAG1* transgenic *Arabidopsis* under wild-type background with 6 rosette leaves curled upwards and misshapen inflorescence. (**C**) early flowering *35S::CyfaAG2* transgenic *Arabidopsis* under wild-type background with 2 rosette leaves curled upwards and misshapen inflorescence.

To further evaluate the functional divergence of *CyfaAG1* and *CyfaAG2*, we attempted to rescue the loss-of-function *Arabidopsis ag-1* mutant using *CyfaAG1* and *CyfaAG2*. *35S::CyfaAG1* and *35S::CyfaAG2* constructs were separately introduced into heterozygote *AG/ag-1 Arabidopsis* to create complementation lines by agrobacterium-mediated transformation. Transgenic *Arabidopsis* mutant-allele lines were identified by dCAPS genotyping (Supplementary Figure S3) and were further confirmed by qRT−PCR. Moreover, sixteen *35S::CyfaAG1* lines under wild-type background and 13 independent *35S::CyfaAG1* lines under homozygous *ag-1* mutant background were obtained, while ten *35S::CyfaAG2* lines under wild-type background and six independent *35S::CyfaAG2* lines under homozygous *ag-1* mutant background were obtained. Furthermore, phenotypes of transgenic *Arabidopsis* lines were assayed in wild-type and homozygous *ag-1* mutant backgrounds to evaluate whether *CyfaAG1* and *CyfaAG2* could substitute for the endogenous *AG* gene in determining stamen and carpel identities, respectively. Either *35S::CyfaAG1* or *35S::CyfaAG2* transgenic *Arabidopsis* under a wild-type background were observed obviously flowering early (Figure 8).

**Figure 9:**
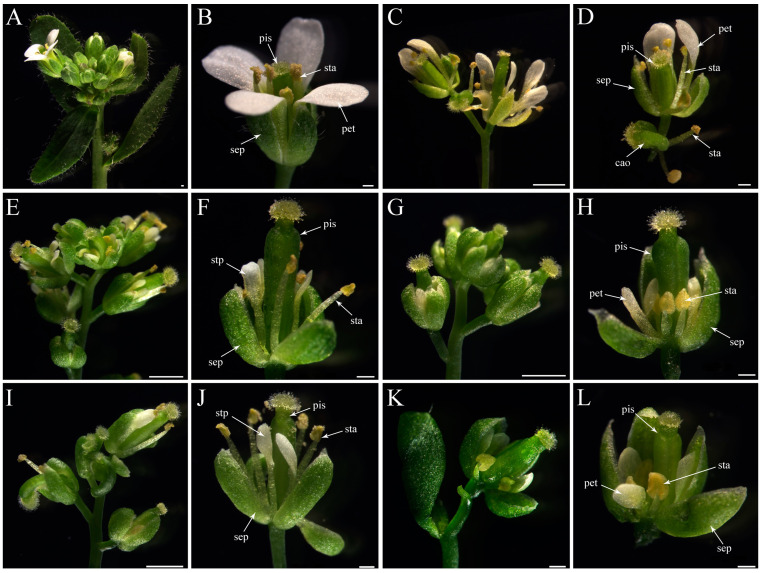
Flower phenotypes comparison of the wild-type, *35S::CyfaAG1* transgenic *Arabidopsis* under wild-type background and *35S::CyfaAG2* transgenic *Arabidopsis* under wild-type background. (**A**) Wild-type *Arabidopsis* with normal raceme; (**B**) normal wild-type *Arabidopsis* flower (four sepals in whorl 1, four petals in whorl 2, six stamens in whorl 3, and pistil in whorl 4); (**C**,**E**,**G**) inflorescence of *35S::CyfaAG1* transgenic *Arabidopsis* under wild-type background; (**D**) flower of *35S::CyfaAG1* transgenic *Arabidopsis* under wild-type background with sepal converted into carpeloid organ and a new flower in the center; (**F**) flower of *35S::CyfaAG1* transgenic *Arabidopsis* under wild-type background with a small sepal in whorl 1, a stamenoid petal (filament with petal at the top) in whorl 2, and a big pistil in whorl 4; (**H**) flower of *35S::CyfaAG1*transgenic *Arabidopsis* under wild-type background with a small sepal in whorl 1, a small petal in whorl 2, small stamens in whorl 3, and a big pistil in whorl 4; (**I**,**K**) inflorescence of *35S::CyfaAG2* transgenic *Arabidopsis* under wild-type background; (**J**) flower of *35S::CyfaAG2* transgenic *Arabidopsis* under wild-type background with a stamenoid petal (filament with petal at the top) in whorl 2; (**L**) flower of *35S::CyfaAG2* transgenic *Arabidopsis* under wild-type background with a small petal in whorl 2, small stamens in whorl 3, and a big pistil in whorl 4. Sepal (sep), petal (pet), filament (fil), anther (ant), stamenoid petal (stp), pistil (pis), carpeloid organ (cao). Scale bars: 500 μm.

Among sixteen *35S::CyfaAG1* transgenic *Arabidopsis* under a wild-type background, one (6.25%) showed a strong phenotype with a sepal converted into a carpeloid organ and a new flower in the center (Figure 9C,D); two (12.50%) displayed a medium phenotype with a small sepal in whorl 1, stamenoid petal (filament with petal at the top) in whorl 2, and a big pistil in whorl 4 (Figure 9E,F); four (25.00%) showed a weak phenotype with a small sepal in whorl 1, a small petal in whorl 2, small stamens in whorl 3, and a big pistil in whorl 4 (Figure 9G,H); and the remaining nine lines (56.25%) had flowers similar to the wild-type *Arabidopsis* flowers.

Among ten *35S::CyfaAG2* transgenic *Arabidopsis* under a wild-type background, four (40.00%) produced flowers with a small sepal in whorl 1, a stamenoid petal (filament with petal at the top) in whorl 2, and a big pistil in whorl 4 (Figure 9I,J); three (30.00%) showed a weak phenotype with a small sepal in whorl 1, a small petal in whorl 2, a small stamen in whorl 3, and a big pistil in whorl 4 (Figure 9K,L); and the remaining two lines (20.00%) had flowers similar to the wild-type *Arabidopsis* flowers.

**Figure 10:**
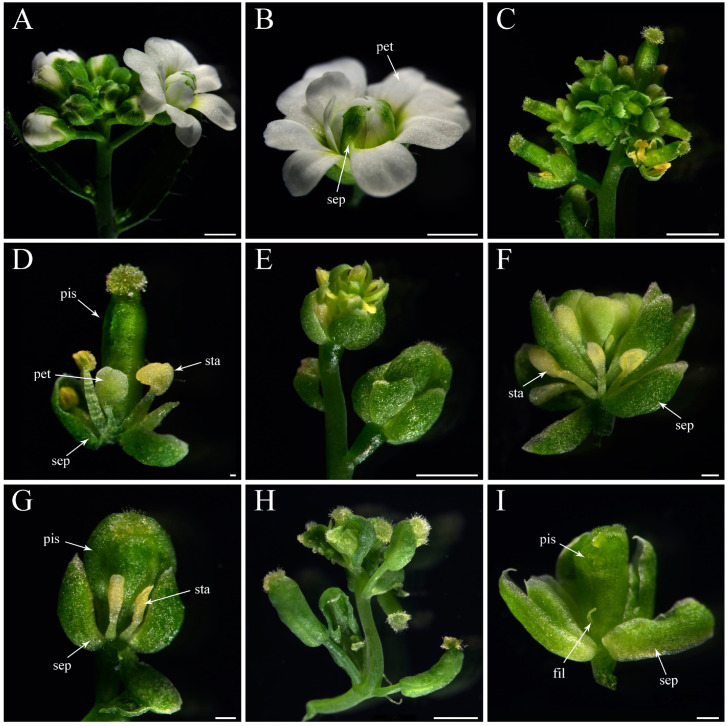
Flower phenotypes comparison of the *Arabidopsis ag-1* mutant, *35S::CyfaAG1* transgenic *ag-1 Arabidopsis,* and *35S::CyfaAG2* transgenic *ag-1 Arabidopsis*. (**A**) Inflorescence of *Arabidopsis ag-1* mutant; (**B**) *Arabidopsis ag-1* mutant flower (sepals in the first whorl, petals in the second and third whorls, and reiterations of this pattern in interior whorls); (**C**) inflorescence of *35S::CyfaAG1* transgenic *ag-1 Arabidopsis*; (**D**) flower of *35S::CyfaAG1* transgenic *ag-1 Arabidopsis* with small petal in whorl 2, stamens in whorl 3, and pistil in whorl 4; (**E**) inflorescence of *35S::CyfaAG1* transgenic *ag-1 Arabidopsis* that consists of a flower with many stamens in whorl 2; (**F**) flower of *35S::CyfaAG1* transgenic *ag-1 Arabidopsis* with sepal in whorl 1, stamen in whorl 2, and reiterations of this pattern in interior whorls; (**G**) flower of *35S::CyfaAG1* transgenic *ag-1 Arabidopsis* with sepal in whorl 1, stamen in whorl 2, and fat pistil in interior whorls; (**H**) inflorescence of *35S::CyfaAG2* transgenic *ag-1 Arabidopsis* that consists of flowers only with sepal and pistil whorls, or filament-like organs and pistil; (**I**) flower of *35S::CyfaAG2* transgenic *ag-1 Arabidopsis* with sepal in whorl 1, filament-like organs in whorl 2, and fat pistil in interior whorl. Sepal (sep), petal (pet), filament-like organ (fil), anther (ant), pistil (pis). Scale bars: 500 μm.

Among thirteen *35S::CyfaAG1* transgenic homozygous *ag-1 Arabidopsis*, one (7.69%) showed a strong complementation phenotype with a sepal in whorl 1, a petal in whorl 2, a stamen in whorl 3, and a pistil in whorl 4 (Figure 10C,D); three (23.08%) displayed a medium phenotype with many stamens in whorl 2 (Figure 10E,F) or sepals in whorl 1, a stamen in whorl 2, and a fat pistil in the interior whorl (Figure 10E,G); the remaining nine lines (69.23%) displayed no complementation and had flowers similar to the flower of the *Arabidopsis ag-1* mutant.

Among six *35S::CyfaAG2* transgenic homozygous *ag-1 Arabidopsis*, one (16.67%) showed a strong complementation phenotype that consisted of flowers only with sepal and pistil whorls, or filament-like organs and pistil (Figure 10H), one (16.67%) produced flowers with a sepal in whorl 1, filament-like organs in whorl 2, and a fat pistil in the interior whorl (Figure 10I), and the remaining four lines (66.67%) displayed no complementation and had flowers similar with the flower of the *Arabidopsis ag-1* mutant.

## 3. Discussion

Orchid reproductive organs (gynostemium) show dramatic changes in morphology compared to the reproductive organs (stamens and carpels) of other angiosperms. In the model species *A. thaliana*, the floral homeotic C-class gene *AG* is responsible for the specification of reproductive organ identity and the control of floral meristem determinacy, as well as the prevention of the misexpression of A-function genes in the reproductive organs [4,5,30]. *AG* expression starts at stage 3 of flower development in the domains of the floral meristem, where stamen and carpel primordia develop, and its expression remains during all stages of stamen and carpel development [11]. Other rosid species *AG* orthologs, such as *PrseAG* from *Prunus lannesiana* [31], *KjAG* from *Kerria japonica* [32] and *EjAG* from *Eriobotrya japonica* [33], were mainly expressed in stamens and carpels, and showed a conserved function for reproductive organ identity determination in core eudicot species. A duplication event of the *AG* lineage in the order Ranunculales, a sister lineage to all other eudicots, results in two functionally redundant but distinguishable *AG*-lineage members [34,35]. *ThtAG1* and *ThtAG2* are two *AG* orthologs from *Thalictrum thalictroides* (Ranunculaceae); *ThtAG1* showed a highly conserved C- function, whereas *ThtAG2* determined ovule identity [35]. Moreover, *NdAG1* and *NdAG2* are *AG* lineage genes from *Nigella damascene* (Ranunculaceae); *NdAG1* confers C-function specifying stamens and carpels identities, while *NdAG2* is involved in regulating carpel development and floral determinacy [36]. Two *AG*-lineage genes, *MawuAG1* and *MawuAG2*, were also found in the basal angiosperm *Magnolia wufengensis*. *MawuAG1* is mainly expressed in stamen, carpel, ovule, and seed, and shows highly conserved C-function in the determination of stamen, carpel, and ovule identity, while *MawuAG2* is mainly expressed in stamen and carpel, and may be only involved in stamen development [37]. All these data suggest that gene duplication led to functional redundancy or sub-functionalization of both *AG* paralogs in eudicots.

In monocot rice, two *AG* lineage genes, *OsMADS3* and *OsMADS58*, redundantly mediate the C-function: *OsMADS3* plays a predominant role in stamen specification, but *OSMADS58* plays a predominant role in floral meristem determinacy and carpel morphogenesis [11]. Three *AG* lineage genes, namely, *ZMM2* and *ZMM23* (both orthologs of rice *OsMADS3*), and *ZAG1* (ortholog of rice *OsMADS58*) were found in *Zea mays* [13,14]. Z*AG1* is expressed early in stamen and carpel primordia, and determines the floral meristem, *ZMM2* is mainly expressed in the anthers and participates in regulating the formation of stamens and carpels [38]. In orchid *C. sinense*, *CsAG1* expressed in the tepals, lips, and gynostemium, and conferred C-function in reproductive organ development, while *CsAG2* was highly expressed in the gynostemium but its function remained unclear [9]. In addition, two *AG*-like genes, *CeMADS1* and *CeMADS2*, were also found in *C. ensifolium* [15]. *CeMADS1* was expressed only in the gynostemium and may have a pivotal C function in reproductive organ development, while *CeMADS2* was expressed in all floral organs and predominantly in gynostemium and its function remained unclear [15].

Here, we have also isolated two *AG*-like genes (*CyfaAG1* and *CyfaAG2*) from *C*. *faberi*. *CyfaAG1* is mainly expressed in root, leaf, anther cap, gynostemium, and ovary, while *CyfaAG2* was expressed only in anther cap, gynostemium, and ovary of *C. faberi*. Moreover, *pCyfaAG1* and *pCyfaAG2* contain CArG-box for MADS-box transcription factor binding and conserved binding sites (AACAAA-/TTTGTT-motif) for floral homeotic APETALA2 transcription factor recognition [16,19]. In addition, both *pCyfaAG1* and *pCyfaAG2* could drive *GUS* to extensively express in stamens and carpels of transgenic *Arabidopsis*. Furthermore, ectopic expressions of *CyfaAG1* and *CyfaAG2* separately promote early flowering phenotypes. Moreover, ectopic expression of *CyfaAG1* produced flowers with sepal converted into carpeloid organ and a new flower in the center or a stamenoid petal (filament with petal at the top) in whorl 2 in wild-type Arabidopsis, while ectopic expression of *CyfaAG2* produced flowers with a stamenoid petal (filament with petal at the top) in whorl 2. Furthermore, complementation of loss-of-function *Arabidopsis ag-1* mutant suggested that *CyfaAG1* could mimic the endogenous *AG* gene to specify stamen and carpel identity. However, ectopic expression of *CyfaAG2* only completes filament and carpel development in the *35S*::*CyfaAG2* transgenic *Arabidopsis ag-1* mutant. Based on these results, it seems that *CyfaAG1* and *CyfaAG2* work redundantly. *CyfaAG1* may confer C-function specifying stamens and carpels identities, as well as floral meristem determinacy, while *CyfaAG2* may only be involved in filament and carpel development. Both genes work together to regulate normal gynostemium and ovary development in *C*. *faberi*. Our data provide more details to understand how C-class function has been partitioned in orchid, and the functions of two *AG*-like genes regulating gynostemium development of *C. faberi*.

## 4. Materials and Methods

### 4.1. Plant Material

*C. faberi* plants were cultivated in redware flowerpots (30 cm × 18.5 cm × 28.2 cm) filled with orchid substrates in the botanical garden of Yangtze University under natural conditions in Jingzhou City, China. Juvenile leaf, sepal, petal, lip, anther, gynostemium, and ovary were separately dissected when the orchid began to bloom, immediately frozen in liquid nitrogen, and stored at −80 °C until used. Moreover, 5 mm (early stages), 8 mm (seasonal dormancy of floral bud), 10 mm (dormancy breaking and further development), 15 mm, 20 mm, and 25 mm long floral buds, and first-day opening flowers were also sampled.

### 4.2. Isolation and Characterization of CyfaAG1 and CyfaAG2 from Cymbidium faberi

Total RNA was extracted from floral buds using an EASYspin Plant RNA Kit (Aidlab China) according to the manufacturer’s protocol. The first-strand cDNA of 3′ RACE was prepared, and then the 3′end cDNA sequences of *CyfaAG1 and CyfaAG2* were separately amplified with gene-specific forward primers 3RGSPAG1F and 3RGSPAG2F (Supplementary Table S1) using the 3-full RACE Core Set Ver. 2.0 kit (TaKaRa, Japan) according to the manufacturer’s protocol, but with 45 s annealing at 59 °C. The forward primers design was referenced the sequence of *AG* orthologs *CsAG2* (Genbank accession numbers: MG021185.1) and *CsAG1* (Genbank accession numbers: MG021184.1) from orchid’s close relative species *C. ensifolium*. In addition, the 5′ end cDNA sequences of *CyfaAG1* were amplified through 5′RACE using the 5′RACE System for Rapid Amplification of cDNA Ends (Invitrogen, Carlsbad, CA, USA) following the manufacturer’s protocol and the gene-specific primers 5RAG1GSP1, 5RAG1GSP2, and 5RAG1GSP3 (Supplementary Table S1). The 5′ end cDNA sequences of *CyfaAG2* were amplified according to the above method, but with the gene-specific primers 5RAG2GSP1, 5RAG2GSP2, and 5RAG2GSP3 (Supplementary Table S1). The phylogenetic tree was constructed by using MEGA version 5.05 with the neighbor-joining (NJ) method. The NJ tree was constructed with 1000 bootstrap replications. All the C-class and D-class MADS-box transcription factors (TF) with complete sequences were obtained from NCBI Genbank (Supplementary Table S2).

### 4.3. Isolation and Sequence Analysis of CyfaAG1 and CyfaAG2 Promoters from Cymbidium faberi

*C*. *faberi* genomic DNA was extracted from juvenile leaves using the CTAB Plant Genomic DNA Rapid Extraction Kit (Aidlab, Beijing, China) according to the manufacturer’s protocol. The *CyfaAG1* 5′ flanking regions were amplified from *C*. *faberi* genomic DNA using the Genome Walking Kit (TaKaRa, Japan) following the manufacturer’s protocol and with gene-specific primers pAG1SP1, pAG1SP2, and pAG1SP3 (Supplementary Table S1) for the first walking sequencing, and with the gene-specific primers pAG1SP4, pAG1SP5, and pAG1SP6 (Supplementary Table S1) for the second walking sequencing. Meanwhile, the *CyfaAG2* 5′ flanking regions were amplified according to the above method, but with the gene-specific primers pAG2SP1, pAG2SP2, and pAG2SP3 (Supplementary Table S1) for the first walking sequencing, and with the gene-specific primers pAG2SP4, pAG2SP5, and pAG2SP6 (Supplementary Table S1) for the second walking sequencing, and the gene-specific primers pAG2SP7, pAG2SP8, and pAG2SP9 (Supplementary Table S1) for the third sequencing. The primers designed for the first walking were separately based on the corresponding gene sequences *CyfaAG1* (Genbank accession number: MH917913) and *CyfaAG2* (Genbank accession number: MH917914). The putative transcription start sites of *CyfaAG1* and *CyfaAG2* were searched through 5′RACE according to the above method. The cis-acting elements located at the *CyfaAG1* and *CyfaAG2* promoter regions were separately searched in the PLACE database [39].

### 4.4. Characterization of pCyfaAG1 and pCyfaAG2 Activity from the 5′ Deleted Promoter Fragments in Transgenic Arabidopsis

We separately designed three forward primers (TpCyfaAG1F, TpCyfaAG1F1, and TpCyfaAG1F2) and a reversed primer TpCyfaAG1R (Supplementary Table S1) to amplify 5′-deletion fragments of *pCyfaAG1*. In addition, we also designed three forward primers (TpCyfaAG2F, TpCyfaAG2F1, and TpCyfaAG2F2) and a reversed primer TpCyfaAG2R (Supplementary Table S1) to amplify the promoter deletions of *pCyfaAG2*, respectively. Three 5′-deletion fragments of *pCyfaAG1* were separately designated as p1D1 (−1207/+185), p1D2 (−1191/+185), and p1D3(−387/+185), and then cloned into the pCAMBIA1300 vector with *Xba* I and *Sac* I using the ClonExpress^®^ Ultra One Step Cloning Kit (Vazyme, Nanjing, China) following the manufacturer’s protocol. In addition, three 5′-deletion fragments of *pCyfaAG2* were separately designated as p2D1 (−1473/+187), p2D2 (−956/+187), and p2D3 (−50/+187), and then cloned into the pCAMBIA1300 vector by using the above method. All the constructs were separately transformed into *A. thaliana Col-0* plants (ecotype Columbia) with the floral-dip method suggested by Clough and Bent [40]. Transgenic Arabidopsis seedlings were selected, cultivated, and prepared for histochemical GUS staining according to Zeng et al. [41]. The expression of the *GUS* gene controlled by different 5′-deletions of *pCyfaAG1* and *pCyfaAG2* were also separately confirmed via qRT−PCR in transgenic Arabidopsis with the primers qGUSF and qGUSR (Supplementary Table S1). An amplification fragment of *A. thaliana Actin* (Genbank accession numbers: AY114679.1) with the primers qActinF and qActinR (Supplementary Table S1) was used as the internal control.

### 4.5. Cytomorphological Examination and Expression Analysis of CyfaAG1 and CyfaAG2

The six stages of floral buds described above and first-day opening flower of *C*. *faberi* were separately sampled and fixed in FAA (38% formaldehyde: acetic acid: 70% ethanol = 1:1:18, by vol.). The samples were dehydrated in an ethanol series (1.5 h each), cleared in a xylene series (2 h each), infiltrated with a xylene and paraffin (12 h at 38 °C) series, followed by three changes of 100% molten paraffin at 60 °C (4 h each), then embedded into a paraffin block, which was serially sectioned at a thickness of 8 µm with a Leica RM2235 rotary microtome after two days. Subsequently, the sections were stained with safranin-fast green [42]. The sections were observed under a CAIKON RCK-40C microscope and photomicrographs were subsequently taken.

The total RNA of each sample was extracted according to the above method, but the first-strand cDNA was synthesized for quantitative real-time PCR (qRT-PCR) by using the HiScript^®^ II Q RT SuperMix for qPCR kit (Vazyme, Nanjing, China) following the manufacturer’s protocol. The relative expressions of *CyfaAG1* and *CyfaAG2* were separately detected in root, juvenile leave, sepal, petal, lip, anther, gynostemium, and ovary of *C. faberi* plants according to Fei and Liu [1], but with the gene-specific forward primer qCyfaAG1F and reverse primer qCyfaAG1R for *CyfaAG1*, and with the gene-specific forward primer qCyfaAG2F and reverse primer qCyfaAG2R for *CyfaAG2*, respectively (Supplementary Table S1). The qRT−PCR was performed with three biological replicates and amplification of *C. faberi* actin (Genbank accession numbers: JN177719.1) fragment was used as the internal control with the forward primer qCyfaactinF and reverse qCyfaactinR (Supplementary Table S1).

### 4.6. Ectopic Expression of CyfaAG1/CyfaAG2 in Arabidopsis ag-1 Mutant and Function Analysis

Full-length *CyfaAG1* cDNAs in the sense orientation were cloned into the pBI121 vector with *Xba* I and *Sma* I restriction enzymes, and the forward primer TCyfaAG1F and the reverse primer TCyfaAG1R (Supplementary Table S1) under control of the CaMV35S promoter using the ClonExpress^®^ Ultra One Step Cloning Kit (Vazyme, Nanjing, China) according to the manufacturer’s protocol. Full-length *CyfaAG2* cDNAs in the sense orientation were cloned into the pBI121 vector according to the above method, but with the forward primer TCyfaAG2F and the reverse primer TCyfaAG2R (Supplementary Table S1). The 35S::*CyfaAG1* and 35S::*CyfaAG2* constructs were separately transformed into heterozygous *Ag/ag-1 Arabidopsis* using the floral-dip method suggested by Clough and Bent [40]. Transgenic *Arabidopsis* seeds were selected and seedlings were subsequently transplanted into soil for cultivation in a growth chamber according to Li et al. [43]. Wild-type, heterozygous *AG/ag-1* and homozygous *ag-1* transgenic *Arabidopsis* lines were confirmed by the dCAPS finder program suggested by Neff et al. [44]. The phenotypes of transgenic *Arabidopsis* were analyzed after flowering. In addition, the complementation degrees of independent transgenic lines of 35S::*CyfaAG1* and 35S::*CyfaAG2* homozygous *ag-1 Arabidopsis* were categorized as ‘no complementation’, ‘weak complementation’, ‘medium complementation’ and ‘strong complementation’, respectively. Moreover, independent transgenic lines of each complementation degree were confirmed by qRT−PCR with the primers qCyfaAG1F and qCyfaAG1R for *CyfaAG1* (Supplementary Table S1), and with the primers qCyfaAG2F and qCyfaAG2R suggested above for *CyfaAG2* (Supplementary Table S1), respectively. An amplification fragment of *A. thaliana Actin* (Genbank accession numbers: AY114679.1) with the primers qActinF and qActinR was used as the internal control.

## 5. Conclusions

Orchid reproductive organs (gynostemium) show dramatic changes in morphology compared to reproductive organs (stamens and carpels) of other angiosperms. In *Arabidopsis*, *AGAMOUS* (*AG*) play roles in determining stamens and carpels identities, floral meristem determinacy, and repression of the A-function. However, the molecular basis of orchid gynostemium development remains largely unknown. In this study, two *AG*-like genes, *CyfaAG1* and *CyfaAG2*, and their promoters were isolated and functionally characterized in a Chinese famous traditional orchid *C. faberi*. Both *CyfaAG1 and CyfaAG2* are highly expressed in the anther cap, gynostemium, and ovary. Moreover, both *pCyfaAG1* and *pCyfaAG2* could drive *GUS* to extensively express in stamens and carpels of transgenic *Arabidopsis*. Ectopic expression of *CyfaAG1 and CyfaAG2* promotes early flowering of wild-type *Arabidopsis*. Moreover, ectopic expression of *CyfaAG1* completely rescues floral defects in the *Arabidopsis ag-1* mutant, while ectopic expression of *CyfaAG2* only completes filament and carpel development. Our findings suggest that *CyfaAG1* acts as an evolutionarily conserved C-function gene in determining reproductive organ identity and mediating floral meristem determinacy. *CyfaAG2* redundantly mediates the C-function in floral meristem determinacy and gynostemium development. Our results provide more details to understand how the C-class function has been partitioned in orchid, and the roles of two *AG* orthologs involved in the gynostemium development of *C. faberi.*

## Data Availability

All data generated or analyzed during this study are included in this published article.

## Supplementary Materials

The following supporting information can be downloaded at: https://www.mdpi.com/article/10.3390/plants12142740/s1. Table S1: Primers used in this study. Table S2: Information on Sequences selected for alignments and phylogenetic analyses from NCBI GenBank. Figure S1: *CyfaAG1* promoter sequence. Figure S2: *CyfaAG2* promoter sequence. Figure S3. Genotyping of wild-type, heterozygote and homozygous ag-1 mutant A. thaliana (Landsberg erecta) plants by dCAPS.
