# Supplementary material for: Characterization of Two AGAMOUS-like Genes and Their Promoters from the Cymbidium faberi (Orchidaceae)
Source: Plants (Basel). 2023 Jul 24;12(14):2740. doi: 10.3390/plants12142740 (PMC10386245; doi:10.3390/plants12142740)
Supplement: Supplementary file 1 [file plants-12-02740-s001.zip › plants-2462195-supplementary.pdf]

Supplementary Materials

Supplementary Table S1: Primers used in this study.

Primer name	Primer sequence (from 5' to 3')
CyfaAG1F	GTCTTCTCAGTCAGGAAAC
CyfaAG1R	GGACTTGAGGAAACTCATC
3RGSPAG2F	ATGGGGAGGGGGAAGATAGAGAT
CyfaAG2F	AGTCGACTATGATGGAGCCAA
CyfaAG2R	ATCTCCATCAATGCAACTCTC
pAG1SP1	TGCATATTCATAAAGGCGACCACGGC
pAG1SP2	CGACCTCAGCATCACAGAGAACA
pAG1SP3	TCTCTTGATCTCTATCTTCCCCCTCC
pAG1SP4	GAACCACCTCTTATTTACACTTCGAG
pAG1SP5	CTCAAAGCAAGCAGCAATGGCCTCA
pAG1SP6	GCCTGAGCTGCACTCACACTAATAT
pAG2SP1	GAGGCTTCTTGCATGTAATACTGCGC
pAG2SP2	CCTTCACACTGTTATTGGCATAC
pAG2SP3	TACAGAAGGTGACTTGCCTGTTC
pAG2SP4	TTGTGCTTGCTGCGCTTCTTCTCC
pAG2SP5	TTAGATGATGGGTGGGCACTAGAG
pAG2SP6	TGGCGTTTGAATACGACGTTGGAC
pAG2SP7	GTTTGAAAGGGCAGCCATGACAG
pAG2SP8	CTAGTTTCACCCACATTCTCGTCG
pAG2SP9	CTTGCAGTTTTACATGGATTAGACC
5RAG1GSP1	ATGCTGATGCTGCTGTGCTC
5RAG1GSP2	CTACGCAGGTACATGTTATCAGTTTGG
5RAG1GSP3	GCATCACCCAGTAAGTTCCTGTTG
5RAG2GSP1	GTTTATGTGTGGCTGCTGT
5RAG2GSP2	CGGAGGTACATGTTGTCGTTTTGCAGT
5RAG2GSP3	CCCATCAGATTCCTGTTTGAGTTC
TpCyfaAG1F	ATGATTACGAATTC<u>GAGCTC</u>CCACCATTTTAATGTTATAGAAA
TpCyfaAG1F1	ATGATTACGAATTC<u>GAGCTC</u>ATAGAAATTATATGAGAACTTCA
TpCyfaAG1F2	ATGATTACGAATTC<u>GAGCTC</u>AGATCAATGGTAGTAGCCAGTTA
TpCyfaAG1R	TCTGCAGGTCGAC<u>TCTAGA</u>GTTTCCTTCCAACAGGAAAAATCAAC
TpCyfaAG2F	ATGATTACGAATTC<u>GAGCTC</u>GTCGAGTTTGGAGTTATAGTTTTAAG
TpCyfaAG2F1	ATGATTACGAATTC<u>GAGCTC</u>GAGTTTTCCTAGCAAACATGAT
TpCyfaAG2F	ATGATTACGAATTC<u>GAGCTC</u>GCTTCAAGACTTATCAGCTTAA
TpCyfaAG2R	TCTGCAGGTCGAC<u>TCTAGA</u>AGTCGACTCCGTTGGTCCTTCC
qGUSF	GTTCTGCGACGCTCACACCGATA
qGUSR	CAGCCATGCACACTGATACTCTTC
qActinF	GACCGTATGAGCAAAGAGATC
qActinR	CACATCTGTTGGAAGGTGCT

qCyfaAG1F	CCTGCGTAGTAAGATAGCTGACAATG
qCyfaAG1R	GCAGTCTGTTGCTGGTGAGAATAATG
qCyfaAG2F	GGGAGATGGAACTGCAAAACGAC
qCyfaAG2R	GCTGTTGGAGAGAATAGTGATGGCTG
qCyfaactinF	AAGACTTACACCAAGCCGAAGAAGATC
qCyfaactinR	CCAGCTCCACAGGTTGCGTTAG

Supplementary Table S2: Information on Sequences selected for alignments and phylogenetic analyses from NCBI GenBank.

Taxon and species		Protein name	Accession numbers	Lineage
Magnoliaceae				
	Nymphaea tetragona	MsAG	ALF02827.1	AG
	Magnolia wufengensis	MawuAG1	QDF82834.1	AG
		MawuAG2	QDF82835.1	AG
		MawuSTK	QDF82836.1	D
Liliaceae				
	Lilium longiflorum	LMADS10	AIJ29174.1	AG
		LMADS2	AAS01766.1	AGL11
Orchidaceae				
	Cymbidium ensifolium	CeMADS1	ADP00515.1	AG
		CeMADS2	ADP00516.1	AG
	Cymbidium faberi	CyfaAG1	QDF44723.1	AG
		CyfaAG2	QDF44724.1	AG
		CfSTK	QDF44725.1	D
	Cymbidium sinense	CsAG1	AVP27263.1	AG
		CsAG2	AVP27264.1	AG
	Dendrobium thyrsiflorum	DthyrAG1	AAY86364.1	AG
		DthyrAG2	AAY86365.1	D
	Dendrobium hybrid cultivar	DOAG1	AXB26295.1	AG
		DOAG2	AXB26296.1	D
	Oncidium hybrid	OMADS2	AIJ29175.1	AGL11
		OMADS4	AIJ29176.1	AG
Asparagaceae				
	Hyacinthus orientalis	HAG1	AAD19360.2	AG
Gramineae				
	Zea mays	ZAG1	AAA02933.1	AG
		ZMM2	AAB81103.1	AG
		ZMM23	CAD23413.1	AG

	Oryza sativa	OsMADS3	Q40704.1	AG
		OsMADS58	BAE54300.1	AG
		OsMADS13	Q2QW53.2	AG
Eupteleaceae				
	Euptelea pleiosperma	EUplAG1	ADC79698.1	AG
		EUplAG2	ADC79699.1	AG
Lardizabalaceae				
	Akebia trifoliata	AktAG1	AAT46102.1	AG
		AktAG2	AAT46096.1	AGL11
Altingiaceae				
	Liquidambar formosana	LAG	AAD38119.1	PLE
Vitaceae				
	Vitis vinifera	Vvmads1	AAK58564.1	PLE
Fabaceae				
	Medicago truncatula	MtAGa	AIT11837.1	euAG
		MtAGb	AIT11838.1	euAG
		MtSHP	AFU81372.1	PLE
Rosaceae				
	Prunus persica	PpMADS4	AAU29513.1	euAG
		PPERSTK	ABQ85556.1	D/AGL11
		PPERSHP	ABG75908.1	PLE
	Prunus lannesiana	PrseAG	ADK95058.1	euAG
		PrseSTK	ADD91578.1	AGL11/D
		PrseSHP	ADG45819.1	PLE
	Geum rupestre	TrAG	ABB59994.1	euAG
		TrSHP	ABB59995.1	PLE
	Rosa rugosa	MASAKO D1	BAA90743.1	PLE
		MASAKO C1	BAA90744.1	euAG
Cucurbitaceae				
	Momordica dioica	MdMADS07	AXR70629.1	euAG
		MdMADS08	AXR70630.1	D
	Cucumis sativus	CUM1	AAC08528.1	euAG
		CUM10	AAC08529.1	AGL11/D
Salicaceae				
	Populus trichocarpa	PTAG1	AAC06237.1	euAG
		PTAG2	AAC06238.1	euAG
	Populus alba	PaAG1	AZR37458.1	euAG

		PaAG2	AZR37457.1	euAG
Brassicaceae				
	Arabidopsis thaliana	AG	P17839.2	euAG
		SHP1	OAP06129.1	PLE
		SHP2	AAU82057.1	PLE
		STK	OAO98566.1	D
Polygonaceae				
	Fagopyrum esculentum	FaesAG	AFO83615.1	euAG
		FaesSTK	QLC27650.1	D
Primulaceae				
	Cyclamen persicum	CpAG1	BAK18552.1	euAG
		CpAG2	BAK18553.1	euAG
		CpSTK	BAK09621.1	STK
Theaceae				
	Camellia japonica	CjAG	AIP87050.1	euAG
Actinidiaceae				
	Actinidia argute	AcarAG	ADU15476.1	euAG
Rubiaceae				
	Coffea arabica	CaAG	AHW58037.1	euAG
Gentianaceae				
	Gentiana scabra	GsAG1	BAS04480.1	PLE
		GsAG2	BAS04484.1	PLE
		GsSTK1	BAS04473.1	D
Solanaceae				
	Nicotiana benthamiana	NbAG	AFK13159.1	euAG
		NbSHP	AFK13160.1	PLE
	Solanum lycopersicum	TAG1	AAA34197.1	euAG
		TAGL11	AAM33102.2	AGL11/D
		TAGL1	AAM33101.2	PLE
	Petunia hybrida	pMADS3	CAA51417.1	euAG
		FBP7	CAA57311.1	D
		FBP11	CAA57445.1	D
		FBP6	CAA48635.1	PLE
Plantaginaceae				
	Antirrhinum majus	FAR	BAI68392.1	euAG
		PLE	BAI68391.1	PLE
Oleaceae				
	Fraxinus pennsylvanica	FpAG	AFP99884.1	euAG

Linderniaceae				
	Torenia fournieri	TfFAR	BAG24494.1	euAG
		TfPLE	BAG24495.1	PLE
Asteraceae				
	Chrysanthemum morifolium	CAG1	BAG06959.1	euAG
		CAG2	BAG06960.1	euAG
		CAG3	BAG06961.1	euAG
	Gerbera hybrida	GAGA1	CAA08800.1	euAG
		GAGA2	CAA08801.1	AG

Supplementary Figure S1: ***CyfaAG1*** **promoter sequence.**

The transcription start site (+1) is in bold and boxed. The start codon ATG is in bold and boxed. Putative cis-acting regulatory elements are in bold and underlined.

> pCyfaAG1

−1207 CCAC**CATTTT AATG**TTAT**AG AAA**TTATATG AGAACTTCAA AAAATAACTT TTCATGT**GTG**
CArG-box POLLEN1LELAT52 GTGANTG10

−1147 **A**TGCTT**TACT** TAACCAAATT TATAAAATAA TTAGCACATA ATTAATATAA AACAAGAGCA
CACTFTPPCA1

−1087 CAAGC**CACT**A TTCATTAGAT TGTATAATTT CTTCCATGAA GATAATAAAA ATCA**TTATTT**
CACTFTPPCA1 TATABOX5

−1027 TATCCCAAAT AAGCTA**TTAT TT**TAATGTTA GAACT**TTTGT T**TATTTTCTA TAAGATTTAA
TATABOX5 TTTGTT motif

−967 GTGCATCCAA CTAAATAATA GAATTAAATT TTTGAAAATT TGGCTATATT TTAGATAAGT

−907 TTTTCTTTCT TAATTGATGA AACTTATAAT ACATAAGAAC TTC**TACT**ATG **AACAAA**GAAT
CACTFTPPCA1 AACAAA motif

−847 TAATAAGTGC TTTTGATCCT ACGAAAAATC GTA**TGAC**A**TG AC**CCCAAAAA TTTAAC**TACT**
WRKY71OS WRKY71OS CACTFTPPCA1

−787 CA**TTATCC**CC AAA**ATTTATA TAAAT**GAAAT TA**CAAT**GCTG AGATCTTAGA TAAAATCTCG
SREATMSD D2GMAUX28 CAATBOX1

−727 A**TGAAAAAT**A AAATTCTAGA GGATGTGGTC ATAGTA**CAAA TG**AT**CATATG** ATCATTCATA
-300ELEMENT MYCCONSENSUSAT MYCCONSENSUSAT

−667 TCATGTCCAC TAGACTAAAA AGATG**CCAAT** ATTCTTGAGC TCAAATGGCA TCGATCTAAA
CCAATBOX1

−607 GATTTACGAT GAAAAAAAGA GC**CCAAT**CAA TCTCGATCGA TCAACATGTC ATCATCAACA
CCAATBOX1

−547 TTAGAGAGAT TGGGTTGGGT CCATGGCCAG AAGTAGGGAG GAATAAATGG CACCTTAGAG

−487 CAAATT**GTGA** ACCACATTGA TCTTAAAATA AATTAGGCAG GGTAGC**CACA TG**GGAGCCAT
GTGANTG10 MYCCONSENSUSAT

−427 ATTCTCTTGT **CATATG**AAGA CAAGTAA**TTA TCC**ATCAAAA AGAT**CAAT**GG TAGTAGCCAG
MYCCONSENSUSAT SREATMSD CAATBOX1

−367 T**TAACCA**AAA TCACCTAGAA TTGTAA**CAAT** ATCAGAATTA GTACCCCCAA AATCTT**AGAA**

MYB1AT CAATBOX1 POLLEN1LELAT52

−307 **A**ATGGCAAGG AACTGAAGGC AAGGGGCTCA AGAGAGAAGT AGGAAGATAA AGGACAATGG

−247 AGCTCTCTCT TCTTTCCCCC TCTCTT**TTTG TT**TTGTATTT TTTTTTCATA GTATATTAGT

TTTGTT motif

−187 **GTGA**GTGCAG CTCAGGCTAG GTCTTTTTCA AACTCCATTA ATAAT**TACT**T TCACAAAGTC

GTGANTG10 CACTFTPPCA1

−127 CCTTT**CAACT C**TC**TCTCTCT CT**CTCTCTCT CTCTCTCTCT CTCTCTCTCA TCTCATCCAT

CAREOSREP1 CTRMCAMV35S

−67 GGAA**GTGA**GG CCATTGCTGC TTGCTTTGAG TTAA**TAACCA** GTCCGTCCTT CTTCTCGAAG

GTGANTG10 MYB1AT

+1

−7 TGTAAAT**A**AG AGGTGGTTCT CAAGT**TATAA AT**TGATAGGC CTCATATCGC TCA**AGAAA**CA

TATABOX2 POLLEN1LELAT52

+54 **GTGA**AATAGA TCG**AGAAA**GC TAAGGAACAT TAATCTTGTG TCTTCTCAGT CAGGTAAGGG

GTGANTG10 POLLEN1LELAT52

+114 AGGTAATAA**A GAAA**AAACAC AGAGCTTGAG AGTTAGATCT CTGATAGTTG ATTTTTCCTG

POLLEN1LELAT52

+174 TTGGAAGGAA AC**ATG**

Supplementary Figure S2: *CyfaAG2* promoter sequence.

The transcription start site (+1) is in bold and boxed. The start codon ATG is in bold and boxed. Putative cis-acting regulatory elements are in bold and underlined.

> pCyfaAG2

−1473 GTCGAGTTTG GAGTTATAGT TTTAAGTTAT CTCT**TGAC**AG TAAATATGTA GCTA**TGAC**AT

WRKY71OS WRKY71OS

−1413 TCATAGCATT ATCTTTATT**G ATA**ACTAATT TGTATCTTTT CTCCATCATA **CAAT**ATACAT

GATABOX CAATBOX1

−1353 AATGGAAATA TTTCACATTC TCAAATCTTC ATCTTTAGCT ATTGTTATAC ACCTCAAGAC

−1293 ACT**CACTTG**A TGTTCTATCT GGCGTTCTAT ATTTACATGA **GATA**TAAGAA TATTAACACG

MYCCONSENSUSAT GATABOX

−1233 AAC**CAACTC**A ATTTCATGTT CTATCTTTTC ATCTTATACA TAATCTAAGA ATGTAATTA**T**

CAREOSREP1

−1173 **GAC**CTTCTAT **TGATT**TTGTA TGTGGCTATT CTTATAA**CAC T**TAAAAT**AAA CCA**CATC**TAC**

WRKY71OS ARR1AT CACTFTPPCA1 MYB1AT CACTFTPPCA1

−1113 **T**ATGCTCTTT ATAATATTTA T**TGATT**TAAT TGAGCTAAAT TAAAATTAAT GTAAT**CAATT**

ARR1AT

−1053 **TTAAG**TCAAA AT**CAAT**TT**TG ATT**ATGAATT AGAACTTGAA ATCAACTTGA AA**TTATTT**GG

CArG-box CAATBOX1 ARR1AT TATABOX5

−993 AGTTGCATTA AATTTTA**AAC AAA**TTTCATG GAATTTTGAG TTTTCCTAGC AAACA**TGATT**

AACAAA motif ARR1AT

−933 CTACACTAGA AATGTGAATA AATTAGAGAA TATATAAACC TATCATTTCT ATAAGTGCTA

POLLEN1LELAT52

−873 AATCTTTCAG AAAGGTCTAA TCCATGTAAA ACTGCAAGAT TAATTATCTT CATGAATGAT

POLLEN1LELAT52 ARR1AT

−813 CCATTTACGG TAAATAAGAA GCAAGTACAC AAAGTTAAAG TAGCAACACA CTTAACGACG

CACTFTPPCA1

−753 AGAATGTGGG TGAAACTAGC CAGCTAATAT GCTTTAGAAG AAAGGACCTG TGATGGTCCC

GTGANTG10

−693 AGGAGATGAT GTTGGCATTT ACGATGATCT GTCATGGCTG CCCTTTCAAA CACCCACTTT

PROXBBNNAPA

−633 GCATTGCAAG CCTGTTATAG CAAAATAAAC TATCTTTAGA GCTCACAGTT CATCTATGGT

MYBCORE

−573 TCTTCGATCT TAACCACAAT TAGATCTCCA AAGAATTTAA GAAAATTGTA CATAATCTTA

MYB1AT POLLEN1LELAT52

−513 TATATCAAAG GCCATGTCAG CTCTAGCATC AGAAAAATTG CTTCCATGGC CTACATTATG

POLLEN1LELAT52

−453 GTGTCAAAAA ACGAATTTTA AAAATACTTA AAGCTTTATG GTTTGCAAAA GTATTTGAAT

-300ELEMENT

−393 TAAGAGTCAA AATATCAGAA AAAAATTAAT GAGCGGAAGA CAAAGGGCCA AGAATTGAAG

POLLEN1LELAT52

−333 AGGGAGTTGA AGGACAATGG CGCACTGCGT GCTTTCATCC TTCATCGTTC CTTTTGTTTT

CAATBOX1 CACTFTPPCA1 PYRIMIDINEBOXOSRAMY1A

−273 ATTTTACATA GTATATTTGT TTGAGTGCAA TTCAGTCCAA CGTCGTATTC AAACGCCATT

TATABOX5 TTTGTT motif CAATBOX1

−213 AATAATCCCT TTCTAAAAGA CCCTTTCCCA TCTTTCTCTC TCTATCTCTT TTCTCTAGCC

CTRMCAMV35S

−153 CTCTTCTCTC TCTAGTGCCC ACCCATCATC TAATCCTTTG AAACCGATGG CAATGGTTGC

CTRMCAMV35S CAATBOX1

−93 CTGAGTTAAT AACCTGTCCT TTCATTGTAG AAAGAGAGGG AAAGCTTCAA GACTTATCAG

POLLEN1LELAT52

+1

−33 CTTAATAACC AGCAAGTTGC TCCCCTTTCC TTCAAAGAAA ACTGGCTTTC TTTCAGGCAG

+28 GAACCGGAGA AGAAGCGCAG CAAGCACAAC TCAATTTGCA GAGTCCGTAA ATTTAATGTT

CAREOSREP1

+88 TCTTCTTTCT CTTCAAAAAG TTGCAATTTT CGATTTCTGA AATTTATTTG TGGCTTTTTT

CAATBOX1 ARR1AT TATABOX5 CArG-box

+148 TTGTTTGTTT TGTTCTGTGG AAGGACCAAC GGAGTCGACT ATG

TTTGTT motif MYB2CONSENSUSAT

Supplementary Figure S3. Genotyping of wild-type, heterozygote and homozygous *ag-1* mutant *A. thaliana* (Landsberg erecta) plants by dCAPS.

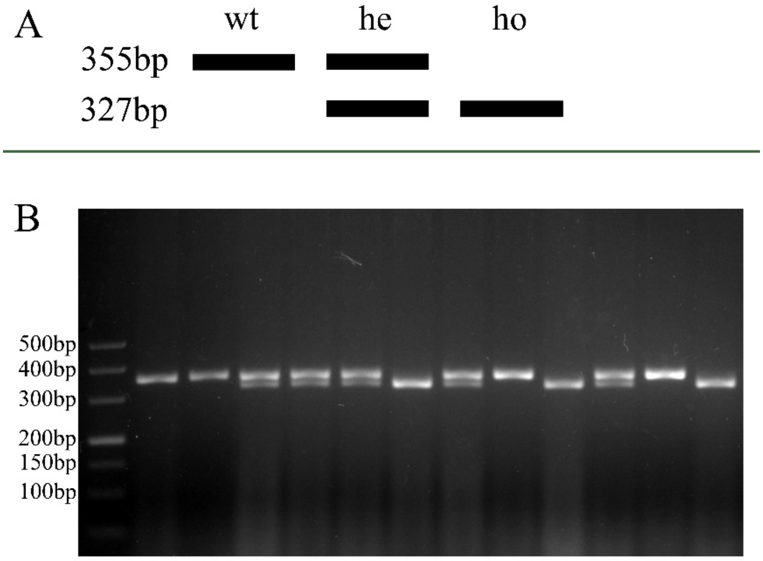

The amplicons of transgenic lines under wild-type background were cleaved By *Afl* II to produce a fragment of 355 bp fragment, the amplicons from transgenic heterozygote *ag-1 Arabidopsis* were cleaved by *Afl* II to produce a 355 bp and a 327 bp fragments, and the amplicons from transgenic homozygous *ag-1 Arabidopsis* were cleaved by *Afl* II to produce a 327 bp fragment.
